# Network Pharmacology Approaches to Myocardial Infarction Reperfusion Injury: Exploring Mechanisms, Pathophysiology, and Novel Therapies

**DOI:** 10.3390/biomedicines13071532

**Published:** 2025-06-23

**Authors:** Joy Das, Ashok Kumar Sah, Ranjay Kumar Choudhary, Rabab H. Elshaikh, Utpal Bhui, Shreya Chowdhury, Anass M. Abbas, Manar G. Shalabi, Nadeem Ahmad Siddique, Raji Rubayyi Alshammari, Navjyot Trivedi, Khoula Salim Ali Buwaiqi, Said Al Ghenaimi, Pranav Kumar Prabhakar

**Affiliations:** 1 School of Pharmaceutical Sciences, Lovely Professional University, Phagwara 144411, Punjab, India; joydas5852@gmail.com (J.D.); bhuiutpal875@gmail.com (U.B.); 2 Department of Medical Laboratory Sciences, College of Applied & Health Sciences, A’ Sharqiyah University, Ibra 400, Oman; rabab.mahmoud@asu.edu.om; 3 Department of Medical Laboratory Technology, UIAHS University Institute of Allied Health Sciences, Chandigarh University, Mohali 140413, Punjab, India; r.choudharymt@gmail.com; 4 Department of Pharmaceutical Sciences, NSHM Knowledge Campus, Durgapur 713212, West Bengal, India; 5 Department of Pharmacy, BGC Trust University Bangladesh, Chittagong 4381, Bangladesh; shreya111727@gmail.com; 6 Department of Clinical Laboratory Sciences, College of Applied Medical Sciences, Jouf University, Sakaka 72388, Saudi Arabia; anasseen@hotmail.com (A.M.A.); dr.mpathology@gmail.com (M.G.S.); 7 Department of Pharmaceutical Chemistry, University of Hafar Al Batin, Hafar Al-Batin 31991, Saudi Arabia; nasiddique@uhb.edu.sa; 8 Department of Pharmacy Practice, University of Hafar Al Batin, Hafar Al-Batin 31991, Saudi Arabia; rralshamari@uhb.edu.sa; 9 Department of Physiotherapy, University Institute of Allied Health Sciences, Chandigarh University, Mohali 140413, Punjab, India; trivedinavjyotphysio@gmail.com; 10 Research, Innovation and Technology Transfer Center, A’ Sharqiyah University, Ibra 400, Oman; 11 College of Applied and Health Sciences, A’ Sharqiyah University, Ibra 400, Oman; 12 Department of Biotechnology, School of Engineering and Technology, Nagaland University, Meriema, Kohima 797004, Nagaland, India

**Keywords:** myocardial infarction, cardiac reperfusion injury, oxidative stress, calcium overload, mitochondrial dysfunction, molecular target, KEGG pathway analysis

## Abstract

Myocardial infarction (MI) remains a leading cause of morbidity and mortality worldwide. While timely reperfusion therapies such as percutaneous coronary intervention (PCI) and thrombolysis are essential for salvaging ischemic myocardium, they can paradoxically exacerbate tissue injury through a process known as myocardial infarction reperfusion injury (MIRI). MIRI can contribute to up to 50% of the final infarct size, significantly diminishing the benefits of revascularization and leading to worsened cardiac outcomes. The pathophysiology of MIRI involves complex, interrelated mechanisms including oxidative stress, calcium overload, mitochondrial dysfunction, inflammatory responses, apoptosis, and dysregulated autophagy. Post-reperfusion recovery is further complicated by structural and functional abnormalities such as microvascular obstruction, endothelial dysfunction, and myocardial stunning. Clinically, distinguishing reperfusion injury from ischemic damage is challenging and often requires the use of sensitive biomarkers, such as cardiac troponins, alongside advanced imaging modalities. Although a range of pharmacological (e.g., antioxidants, calcium channel blockers, mitochondrial stabilizers, anti-inflammatory agents) and non-pharmacological (e.g., hypothermia, gene therapy, stem cell-based therapies) interventions have shown promise in preclinical studies, their clinical translation remains limited. This is largely due to the multifactorial and dynamic nature of MIRI. In this context, network pharmacology offers a systems-level approach to understanding the complex biological interactions involved in MIRI, facilitating the identification of multi-target therapeutic strategies. Integrating network pharmacology with omics technologies and precision medicine holds potential for advancing cardioprotective therapies. This review provides a comprehensive analysis of the molecular mechanisms underlying MIRI, examines the current clinical challenges, and explores emerging therapeutic strategies. Emphasis is placed on bridging the translational gap through validated, multi-target approaches and large-scale, multicenter clinical trials. Ultimately, this work aims to support the development of innovative and effective interventions for improving outcomes in patients with myocardial infarction.

## 1. Introduction

Cardiovascular diseases (CVDs) continue to be the leading causes of mortality and morbidity worldwide, and myocardial infarction (MI), also referred to as a heart attack, is a major contributor. In MI, the blood flow to a part of the myocardium is suddenly stopped or ceased as a result of the rupture of an atherosclerotic plaque within the coronary arteries with thrombus formation. This interruption in blood flow leads to ischemia and, if left untreated, leads to irreversible myocardial cell death. Of all causes, coronary heart disease represents the leading cause of MI in the United States and accounted for 371,506 deaths in 2022 among adults aged 20 and over (ca. 5%) [1]. The management of MI includes timely restoration of blood flow, also called reperfusion. Reperfusion therapies, such as percutaneous coronary intervention (PCI) and thrombolytic therapy, are used to revascularize the ischemic myocardium as soon as possible in order to reduce infarct size and improve the clinical outcome. In particular, PCI is now the reperfusion strategy of choice because it is effective at quickly restoring coronary blood flow and reducing mortality [2]. Nonetheless, paradoxically, reperfusion itself can trigger further damage to the myocardium (myocardial ischemia reperfusion injury, or MIRI), which severely reduces the beneficial impact of reperfusion therapies used in the treatment of MI. In experimental models, MIRI can account for up to 50% of the final infarct size, demonstrating its clinical significance. MIRI is the outcome of highly intricate cellular and molecular mechanisms, which involve oxidative stress, calcium overload, mitochondrial dysfunction, and inflammatory responses [3]. The excessive production of ROS, a consequence of reperfusion, causes lipid peroxidation, protein modification, and DNA damage, resulting in cell dysfunction and death. Furthermore, prior to reperfusion, calcium homeostasis is further disrupted during ischemia and activates pathways that favor cardiomyocyte injury even more deleteriously during reperfusion [4]. Moreover, additional inflammatory responses intensify further tissue damage through the induction of neutrophil activation and the release of cytokines, as well as mitochondrial permeability transition pore (mPTP) opening, leading to ATP depletion and apoptosis. These culminate in structural and functional abnormalities, including microvascular obstruction (no-reflow phenomenon), endothelial dysfunction, myocardial stunning, and irreversible cell death [5].

However, clinically, it is difficult to separate irreversible ischemic damage from reperfusion injury, which is important for choosing the best therapeutic strategy. Myocardial injury can be assessed using such biomarkers as cardiac troponins and imaging modalities, including magnetic resonance imaging (MRI) and echocardiography, and these can be used to inform clinical decision-making. However, these methods are not sufficiently discriminative to distinguish between infarction-induced damage and reperfusion injury. Pharmacological and non-pharmacological strategies currently used to mitigate MIRI have been included. Pharmacological approaches involve the use of antioxidants that scavenge ROS, calcium channel blockers that prevent calcium overload, and anti-inflammatory agents that attenuate the inflammatory response. Recent efforts have focused on remote ischemic conditioning, whereby a brief episode of ischemia is induced in remote tissues to induce cardioprotection [6]. Hypothermia and other non-pharmacological interventions attempt to reduce metabolic demand and prevent further injury; emerging therapies, including gene therapy and stem cell-based interventions, are being investigated for myocardial repair and regeneration. However, despite large amounts of research, there is a lack of effective translation of these therapeutic strategies into clinical practice. There is also a challenge posed by patient heterogeneity, comorbid conditions, and the complexity of the MIRI pathophysiology. Personalized medicine approaches that use integrative omics technologies (genomics, transcriptomics, proteomics) are being investigated to identify novel therapeutic targets and tailor interventions [7].

This study is essential as it addresses a critical and persistent gap in cardiovascular medicine, namely how to safeguard the heart not only from ischemia, but also from the injurious effects of reperfusion. MIRI remains a significant clinical challenge, with its pathogenesis rooted in a complex interplay of mechanisms, including oxidative stress, calcium overload, mitochondrial dysfunction, inflammation, and regulated cell death pathways such as apoptosis and necroptosis. A thorough and integrated review of these mechanisms is vital for identifying precise molecular targets that may lead to effective therapeutic interventions. Although various experimental strategies, such as ischemic preconditioning, novel pharmacological agents, and gene-based therapies, have demonstrated encouraging results in preclinical studies, their clinical translation has been limited. This underscores the urgent need for continued exploration into innovative and combination therapies that can reliably mitigate MIRI while minimizing adverse effects. The relevance of this study extends beyond the myocardium, particularly in the context of cerebral cardiovascular medicine. The heart–brain axis plays a crucial role in post-infarction recovery and long-term functional outcomes, highlighting the systemic implications of MIRI. A deeper understanding of this interconnection could pave the way for interdisciplinary and integrative treatment approaches. By offering a comprehensive evaluation of the current knowledge, unresolved challenges, and emerging therapeutic strategies, this review aims to support clinicians, researchers, and biomedical scientists in advancing the field. It reinforces the imperative for ongoing innovation and collaboration to develop more precise, effective, and patient-centered therapies. Ultimately, the insights provided in this study aim to contribute meaningfully to improving clinical outcomes for individuals affected by myocardial infarction and its associated complications.

## 2. Pathophysiology of Reperfusion Injury

**Figure 1:**
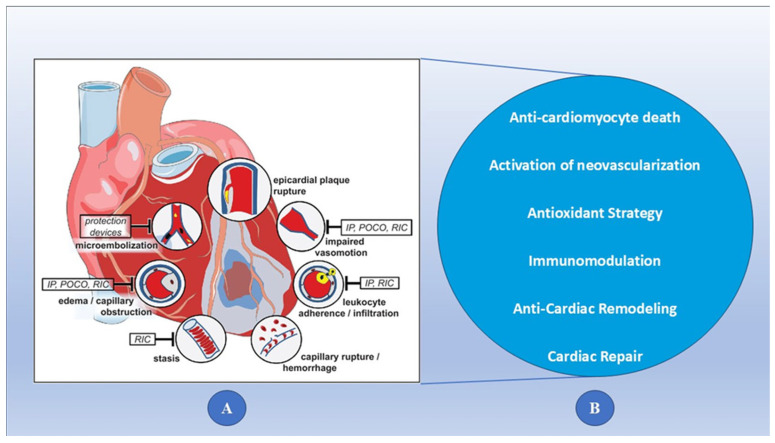
(**A**). Schematic diagram of the pathophysiology of reperfusion injury (**B**). Schematic illustration of therapeutic strategies of myocardial infarction reperfusion injury.

Ischemia reperfusion injury, also termed reperfusion injury, is the paradoxical physiological tissue injury that occurs when blood supply returns to a tissue that had been ischemic for a period of time. This sudden restoration of oxygen and nutrients is the principal cause of this phenomenon, as it can provoke a cascade of inflammatory reactions and oxidative stress. During ischemia, cells undergo metabolic changes in order to deal with the lack of oxygen; when reperfused, the sudden re-entry of oxygen can lead to the generation of ROS and damage to cellular components [8]. Furthermore, reintroduction of the blood flow can cause microvascular injury that includes increased permeability and inflammation caused by white blood cell infiltration into the damaged area and the release of inflammatory mediators. However, each of these factors then contributes to making cells even more dysfunctional and tissue more damaged, further complicating their recovery. The pathophysiology of reperfusion injury was a key step toward the development of therapeutic strategies that may be able to prevent the adverse effects of reperfusion injury in clinical interventions, including organ transplantation and cardiac resuscitation [9]. As illustrated in Figure 1, panel A depicts the pathophysiological mechanisms underlying reperfusion injury, while panel B highlights various therapeutic strategies aimed at mitigating myocardial infarction-related reperfusion injury.

**Figure 2:**
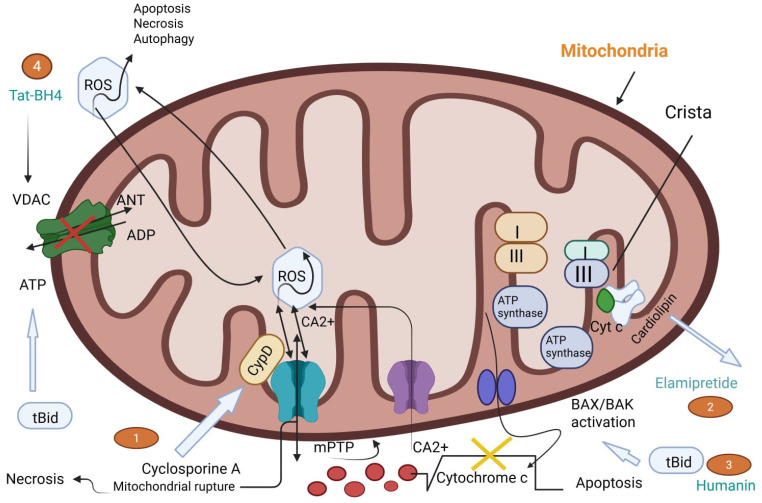
Illustration of mitochondria-mediated apoptosis and therapeutic peptide actions during myocardial ischemia reperfusion injury (MIRI). During acute MIRI, the surge in reactive oxygen species (ROS) and mitochondrial Ca^2+^ overload trigger regulated cell death (RCD) by promoting mitochondrial permeability transition pore (mPTP) opening, leading to apoptosis or necrosis. Elevated ROS levels cause a significant disruption in mitochondrial structure and metabolism, impairing normal mitochondrial function. The diagram highlights the key therapeutic peptides (shown in green) that mitigate intrinsic apoptosis during MIRI, including (1) cyclosporine A (CsA), (2) elamipretide, (3) humanin, and (4) Tat-BH4. Abbreviations: CypD—cyclophilin D; NNT—nicotinamide nucleotide transhydrogenase; FAO—fatty acid β-oxidation; Prx—peroxiredoxins; Gpx—glutathione peroxidase; GsR—glutathione reductase; Trx—thioredoxin; TrxR—thioredoxin reductase; GSH—glutathione; GSSG—oxidized glutathione; PNC—purine nucleotide cycle; tBid—truncated Bid protein; BAX—BCL2-associated X protein; BAK—BCL2 antagonist/killer; OMM—outer mitochondrial membrane; IMM—inner mitochondrial membrane.

MIRI is defined as a series of complex cellular and molecular events that accelerate myocardial damage in comparison with that which can be caused by ischemia alone. MIRI occurs as a result of a number of interconnected mechanisms, including oxidative stress, calcium overload, the inflammatory response, mitochondrial dysfunction, and programmed cell death pathways, including autophagy and apoptosis. Overall, these processes contribute to structural and functional myocardial tissue impairment following reperfusion [10]. An illustration of mitochondria-mediated apoptosis and therapeutic peptide actions during myocardial ischemia reperfusion injury (MIRI) is highlighted in Figure 2.

### 2.1. Cellular and Molecular Mechanisms

#### 2.1.1. Oxidative Stress: Role of ROS in Tissue Damage

The major driver of MIRI is oxidative stress, through which there is excessive production of reactive oxygen species (ROS) during reperfusion. The oxygen supply suddenly restored to ischemic tissue initiates a sudden burst of ROS generation from a multitude of sources, including mitochondria, nicotinamide adenine dinucleotide phosphate (NADPH) oxidases, and xanthine oxidase. The ROS include superoxide anions (O_2_^−^), hydrogen peroxide (H_2_O_2_), and hydroxyl radicals (•OH), which are involved in lipid peroxidation, protein oxidation, and DNA damage, thus causing cellular dysfunction and death. It was reported that mitochondrial electron transport chain dysfunction during reperfusion increases ROS production greatly [11]. In the presence of reverse electron transfer in complex I of the mitochondrial respiratory chain upon reperfusion, there is a surge of superoxide production. Additionally, xanthine oxidase activation due to hypoxia-induced ATP depletion further amplifies ROS levels, exacerbating tissue injury. Elevated ROS also impair nitric oxide (NO) bioavailability, leading to endothelial dysfunction and microvascular injury, which further reduces myocardial perfusion despite revascularization [12].

Although rapid reintroduction of oxygen during reperfusion has been recognized as causing a burst of ROS, including superoxide and hydroxyl radicals produced by mitochondria, NADPH oxidases, and xanthine/xanthine oxidase pathways, more recent research has suggested new dimensions and potential therapeutic options in MIRI. One important mechanistic development relates to ferroptosis, a controlled type of cell death caused by iron-dependent peroxidation of lipids: ischemia speeds up iron release and lipid peroxidation, and reoxygenation further increases ROS, leading to ferroptotic cardiomyocyte death. Notably, small molecules such as liproxstatin-1 or deferoxamine, which can chelate iron and maintain GPX4 activity, have provided strong protection in recent in vivo MIRI models [13,14]. Moreover, recent studies have suggested that ferroptosis, a type of iron-dependent lipid peroxidation, is an alternative damage pathway, which is associated with decreased GPX4 activity and enhanced ROS-mediated lipid oxidation in the reperfusion period [15]. More recent research utilizing bioinformatics platforms has revealed the existence of important oxidative stress-associated genes (OSRGs) such as Hmox1, Gpx7, and Arg1, which adjust antioxidant defenses and inflammatory signaling in MIRI [16]. Another emergent insight is the association between mitochondrial reverse electron transport (RET) and the ROS burst. During ischemia, accumulated succinate can couple RET at complex I upon reperfusion, generating more superoxide than the usual forward flux. Interestingly, pH modulation could attenuate RET-mediated ROS bursts, thus suggesting that the fine regulation of reperfusion acidity may represent an intervention strategy. In addition, ROS-sensitive biomaterials, including nanoparticles or hydrogels that are designed to degrade or release therapeutic agents in high-ROS microenvironments, are increasingly showing marked cardioprotection in animal models of MIRI by targeting the site of injury and delivering sustained antioxidant or anti-inflammatory therapy [14]. In terms of network pharmacology, different polyphenols and flavonoids (resveratrol, naringenin, salidroside) have been indicated, using multi-target analyses and docking simulations to suppress ROS-related signaling (such as FOXO, PI3K-Akt, and NF-kB/TLR4 pathways), stimulate antioxidant defenses (via the Nrf2/HO-1/GPX4 pathways), or even inhibit ferroptosis by upregulating GPX4 and SLC7A11. These network-based interventions frequently take action on many nodes simultaneously, inhibiting ROS, ferroptosis, and inflammation simultaneously [17].

#### 2.1.2. Calcium Overload: Dysregulated Calcium Homeostasis Leading to Myocyte Injury

During ischemia, cellular ATP depletion leads to dysfunction of ion transporters, including Na+/K+ ATPase and the sodium–calcium exchanger (NCX), resulting in intracellular sodium accumulation. Upon reperfusion, the sudden restoration of oxygen and ATP production causes an influx of calcium into cardiomyocytes through the NCX in the reverse mode. This calcium overload triggers hyper-contracture of myocytes, mitochondrial damage, and activation of calcium-dependent proteases such as calpains, further promoting cardiomyocyte death [18]. Calcium overload also leads to the activation of phospholipases and proteolytic enzymes, which degrade membrane phospholipids and cytoskeletal proteins, causing structural instability and rupture of the sarcolemma. Experimental studies have demonstrated that inhibiting calcium influx or enhancing calcium handling through pharmacological agents, such as calcium channel blockers, can mitigate MIRI by reducing intracellular calcium accumulation and preventing cell death [19].

In addition to the traditional story of ATP depletion inhibiting Na+/K+-ATPase and the NCX, recent studies have revealed a more sophisticated picture—particularly regarding the manner by which cardiomyocytes handle post-reperfusion Ca^2+^ bursts. Alongside the NCX and L-type calcium channels, SERCA impairment and abnormal opening of ryanodine receptors (RyR2) play a major role in overwhelming cytoplasmic Ca^2+^ concentrations. New research also highlights store-operated calcium entry (SOCE) via STIM/Orai channels and lysosomal Ca^2+^ release as auxiliary yet previously unrecognized sources of calcium dysregulation [20]. Moreover, calpain, a protease previously well-recognized as a downstream effector of Ca^2+^ overload, now emerges as a master regulator of programmed cell death (PCD) pathways, such as apoptosis, autophagy-dependent cell death, necroptosis, and parthanatos. High levels of calpain activation on reperfusion coordinate the permeabilization of the mitochondrial membrane, cytoskeleton disruption, and signaling cascades of PCD, and inhibition of calpain in preclinical models now demonstrates robust cardioprotective benefits [21]. From a network pharmacology perspective, multi-component TCM-derived formulas (e.g., Danshen, Guanglou Xiebai Decoction) are being discovered to have Ca^2+^ overload attenuating effects by modulating various nodes: upregulating SERCA function through PI3K/AKT/NRF2, RyR2 stabilization, intracellular Ca^2+^ buffering, and inhibition of calpain-mediated downstream PCD. The net effect of these inputs is the reduction of intracellular Ca^2+^ bursts, maintenance of sarcolemma integrity, and inhibition of cell death [22].

#### 2.1.3. Inflammatory Response: Activation of Neutrophils, Cytokines, and Endothelial Dysfunction

Inflammation plays a central role in MIRI, driven by an innate immune response upon reperfusion. The abrupt reoxygenation activates endothelial cells, triggering the release of pro-inflammatory cytokines, such as tumor necrosis factor-alpha (TNF-α), interleukin-1 beta (IL-1β), and interleukin-6 (IL-6). These cytokines stimulate the recruitment and activation of neutrophils, which adhere to the vascular endothelium and transmigrate into the myocardium, exacerbating injury [23]. The release of proteolytic enzymes, myeloperoxidase, and ROS from neutrophils contributes to myocardial damage resulting in further oxidative stress as well as endothelial dysfunction. Additionally, neutrophil extracellular traps (NETs) formation has been associated with the amplification of inflammatory injury and microvascular obstructions. Impaired NO production and upregulation of adhesion molecules such as P-selectin and intercellular adhesion molecule 1 (ICAM-1) enhance leukocyte adhesion and infiltration into the myocardium, demonstrating endothelial dysfunction. The exacerbation of this inflammatory cascade further harms the microvascular unit, further limiting myocardial salvage despite successful epicardial revascularization in the setting of the no-reflow phenomenon [24].

Systemic NET release activates and potentiates myocardial injury in MIRI models and is a contributor to the no-reflow phenomenon; inhibition of NETosis with DNase therapy or PAD4 inhibitors or antioxidant agents significantly decreases tissue injury and microvascular blockages. Moreover, in addition to the traditional cytokine-mediated endothelial activation (e.g., P-selectin, ICAM-1 upregulation), MIRI-induced ROS and NETs work in synergy to impair NO signaling, which triggers the endothelial dysfunction and leukocyte adhesion [25]. In terms of network pharmacology, multi-target anti-inflammatory effects have been reported with natural products such as resveratrol and salvianolic acids and with multi-component Chinese herbal formulas (e.g., total salvianolic acid injection). In addition to dampening the secretion of TNF-alpha, IL-6, IL-1 beta, and IL-18, they also prevent neutrophil activation, NET formation, and NLRP3 inflammasome assembly through the regulation of the PI3K/Akt pathway, the NF-kappa B pathway, and the HIF-1 pathway. Simultaneously, delivery systems, e.g., ROS- and endothelium-responsive nanocomplexes, can co-deliver anti-cytokine siRNAs, antioxidants, and NET inhibitors to injured vasculature, which can limit neutrophil infiltration and NET deposition efficiently [26]. Even recent single-cell analyses revealed the existence of specific neutrophil subpopulations, such as a cardioprotective Ym 1+ subset, which indicates that therapeutic approaches could be used to selectively inhibit pro-inflammatory neutrophils without affecting reparative phenotypes [27]. In the meantime, novel pharmacological therapies, including colchicine, limit neutrophil growth, microvascular occlusion, and NETogenesis after reperfusion [28].

#### 2.1.4. Mitochondrial Dysfunction: Opening of the Mitochondrial Permeability Transition Pore (mPTP)

**Figure 3:**
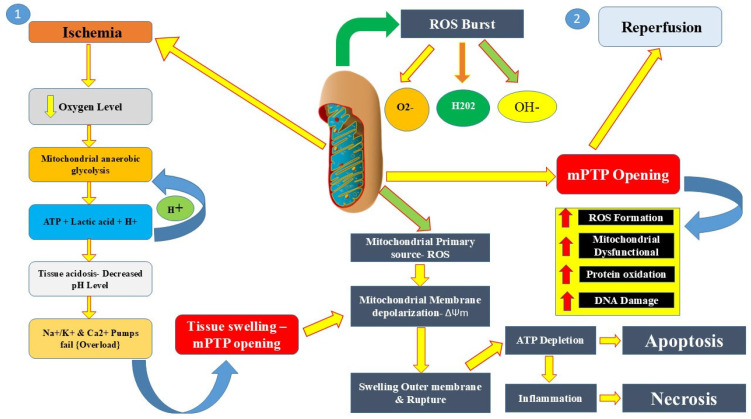
ROS cascade and mPTP opening in myocardial infarction reperfusion injury. (**1**) Ischemia induces low oxygen tension, which causes metabolism to favor anaerobic glycolysis, leading to lactic acid build-up, cellular acidity, and ionic pump failure. This causes tissue swelling and opening of the mitochondrial permeability transition ore (mPTP). (**2**) During reperfusion, the abrupt entry of oxygen forms a burst of ROS, leading to mitochondrial membrane depolarization, outer mitochondrial membrane rupture, and additional opening of mPTP. The ultimate outcomes of these events are ATP depletion, inflammation, and the activation of apoptotic and necrotic pathways of cell death.

A hallmark of MIRI is mitochondrial dysfunction, including the opening of the mitochondrial permeability transition pore (mPTP), and mPTP opening is an important event in cell death. The mPTP is a nonselective, high-conductance channel spanning the inner mitochondrial membrane. Elevated ROS and calcium overload upon reperfusion trigger mPTP opening, followed by mitochondrial swelling, rupture of the outer mitochondrial membrane, and release of pro-apoptotic factors such as cytochrome c into the cytosol [5]. Opening of the mPTP induces dissipation of the mitochondrial membrane potential, decreases ATP synthesis, and leads to the starting of necrotic and apoptotic pathways. Reductions in MIRI have been shown pharmacologically with the use of pharmacological inhibitors of the mPTP, including cyclosporine A, which prevents mitochondrial collapse and preserves cardiomyocyte viability [29]. ROS cascade and mPTP opening in myocardial infarction reperfusion injury are demonstrated in Figure 3.

#### 2.1.5. Autophagy and Apoptosis: Cell Death Pathways in Reperfusion Injury

During MIRI, the death of the cell is caused both by apoptotic and autophagic pathways. There are two main pathways of apoptosis, or programmed cell death: the intrinsic (mitochondrial) pathway and the extrinsic (death receptor) pathway. When mitochondrial cytochrome c is released, this intrinsic pathway is triggered and activates caspase-9 and executioner caspases (caspase-3, caspase-7) to induce DNA fragmentation and cell death. In addition, there is also involvement of the Fas receptor and the extrinsic pathway with TNF-α activation during the reperfusion phase, which promotes apoptosis further [30]. In MIRI, autophagy has a dual role as a process for degrading damaged cellular components. Autophagy at basal levels is cardioprotective through the clearance of damaged and misfolded proteins and mitochondria, but excessive or dysregulated autophagy during reperfusion further compromises cardiomyocytes. Overactivation of autophagy has been demonstrated to result in an excessive loss of essential cellular components and eventually cell death. It has therapeutic potential in reducing autophagy activation in MIRI, and it may be achieved through targeting this process by pharmacologically manipulating it [31].

More recent studies emphasize the importance of redox balance in the orchestration of autophagy and apoptosis during reperfusion. The ARE/Nrf2 axis stands out as a major antioxidant defense system. Nrf2 activation maintains mitochondrial integrity, limiting excessive autophagy and inhibiting apoptotic signaling cascades. Bardoxolone methyl, a strong Nrf2 agonist, was found to lessen infarct size and enhance mitochondrial functioning in rat and mouse models of myocardial ischemia/reperfusion injury (MIRI) [32,33]. Moreover, a natural Nrf2 activator, sulforaphane (SFN), attenuates cardiac cell death by promoting histone acetylation at the Nrf2 promoter, inhibiting HDACs, and shielding against Ang II-mediated cardiomyocyte apoptosis—findings confirmed by in vitro and in vivo experiments [34]. On the other hand, the NLRP3 inflammasome mediates harmful inflammation in reperfusion injuries. NLRP3 inflammasome activation by ROS and mitochondrial DAMPs mediates caspase-1-dependent pyroptosis and promotes myocardial injury. Pharmacological inhibition with MCC950 (also referred to as CP-456,773) markedly reduces infarct size and improves contractile function following I/R in mouse models [35,36]. In transplantation research, normothermic perfusion using MCC950 alleviated reperfusion injury in donor hearts, showing its translational potential [37]. Cell death pathways are also highly regulated by metabolic stress through GLUT transporters. Research has found that activation of DRD4 potentiates GLUT4 translocation through PI3K/AKT to promote glucose uptake and inhibit apoptosis in cardiomyocytes subjected to ischemia-reperfusion injury. In addition, a recent study revealed that PKD1 signaling is a regulator of GLUT4 mobilization, providing a potential target to stabilize cardiac metabolism during reperfusion [38]. The cell fate following reperfusion is also determined by epigenetic mechanisms. Myocardial I/R injury induced by hyperglycemia is reduced by HDAC inhibitors such as TSA or magnesium valproate, which inhibit intrinsic apoptotic pathways [39]. Non-coding RNAs also have crucial roles: miR-144 downregulates Nrf2, while miR-22 interrupts the mitochondrial protection through SIRT1–Nrf2 signaling, augmenting oxidative stress and apoptosis. These mechanistic observations make epigenetic therapies (targeting HDACs, DNA methylation pathways, and microRNAs) novel approaches to regulate oxidative stress, inflammation, autophagy, and apoptosis in MIRI [40].

### 2.2. Hemodynamic and Structural Changes

After myocardial infarction (MI), reperfusion therapy is crucial for restoring the blood flow to the residual infarct tissues but paradoxically aggravates hemodynamic and structural alterations in the infarcted myocardium that impair myocardial recovery. Concomitant with these changes, myocardial stunning, a decrease in the force of contraction, as well as microvascular obstruction (MVO) and endothelial dysfunction leading to capillary leakage all contribute to impaired cardiac function after reperfusion [41].

#### 2.2.1. Microvascular Obstruction (No-Reflow Phenomenon)

Microvascular obstruction (MVO), also known as the no-reflow phenomenon, is the failure of coronary reperfusion to adequately restore perfusion at the microvascular level, despite successful epicardial artery recanalization. This phenomenon is a major determinant of infarct size and a risk factor for poor clinical outcomes, including an elevated risk of heart failure and adverse ventricular remodeling. MVO is a multifactorial pathophysiology in which intravascular, endothelial, and extravascular components play an important part [42]. Capillary occlusion occurs through the plugging of microvascular platelets, leukocytes, and fibrin-rich thrombi, intravascularly. It is known through studies that activated neutrophils participate in a significant part by adhering to the endothelium and forming aggregates, which, as a result, further decreases capillary perfusion. In addition, excessively produced ROS promote endothelial injury and contribute to platelet aggregation and an increase in microvascular resistance [43]. There is also a significant contribution of endothelial dysfunction to MVO. Endothelial swelling resulting from ischemia-reperfusion disrupts the microvascular architecture and impairs capillary patency. Endothelial nitric oxide (NO) bioavailability loss further contributes to vasoconstriction, promotes leukocyte adhesion, and worsens perfusion defects. Extravascular mechanisms include interstitial edemas and hemorrhage, which exert compressive forces on the microvasculature and again restrict the blood flow. During reperfusion, cardiomyocyte swelling leads to capillary collapse, which is indeed confirmed by intravital microscopy studies showing hypoperfusion persisting despite open coronary arteries. In addition, continued inflammation results in sustained microvascular dysfunction, elevating myocardial injury [44].

#### 2.2.2. Endothelial Dysfunction and Capillary Leakage Materials and Methods

Capillary leakage and increased vascular permeability after reperfusion, which are thought to represent a cascade of events leading to MI, are initiated by endothelial dysfunction after reperfusion. Oxidative stress and inflammatory mediators disrupt the endothelial barrier, leaving it vulnerable to plasma extravasation and interstitial edemas through direct plasma molecule and nanoparticle passage and passive diffusion through permeable intercellular tight junctions and adherens junctions [41]. Endothelial activation is caused by an inflammatory cascade triggered by reperfusion, including such cytokines as tumor necrosis factor alpha (TNF-α), interleukin 1 beta (IL-1β), and interleukin 6 (IL-6). Increased expression of adhesion molecules, such as intercellular adhesion molecule-1 (ICAM-1) and vascular cell adhesion molecule-1 (VCAM-1), results in the facilitation of leukocyte infiltration as a result of this process. Adhesion causes leukocytes, most notably neutrophils, to degranulate, releasing proteolytic enzymes and ROS that themselves disrupt endothelial integrity. Under these circumstances, capillary leakage is an important consequence of endothelial dysfunction and thus contributes to interstitial and intracellular edemas [45]. Microvascular dysfunction leads to increased hydrostatic pressure, causing fluid extravasation and resulting in myocardial edemas that subsequently derange contractility and increase myocardial stiffness. Since studies have shown a strong correlation between hyperpermeability of the endothelium, myocardial edemas, and adverse left ventricular remodeling in post-MI patients, studies using contrast-enhanced cardiac magnetic resonance imaging (CMR) have been conducted [46].

#### 2.2.3. Myocardial Stunning and Contractile Dysfunction Materials and Methods

Myocardial stunning is defined as the transient loss of contractile function that persists after reperfusion despite the absence of irreversible myocyte necrosis. This phenomenon is reversible but can significantly impact cardiac output, predisposing patients to heart failure. The mechanisms underlying myocardial stunning involve oxidative stress, calcium dysregulation, and mitochondrial dysfunction. During ischemia, ATP depletion and ionic imbalances disrupt excitation–contraction coupling. Upon reperfusion, a surge in ROS production further impairs calcium-handling proteins, such as the sarcoplasmic reticulum Ca^2+^-ATPase (SERCA2a), leading to calcium overload [47]. Excess intracellular calcium also activates proteases that destroy contractile proteins and thus diminish myocardial contractility. Myocardial stunning is also due to mitochondrial dysfunction. Upon reperfusion, restoration of oxygen occurs rapidly with leakage leading to mPTP opening and concomitant dissipation of the mitochondrial membrane potential, depletion of ATP, and failure of energy production. Pharmacological inhibition of mPTP opening has been shown to reduce myocardial stunning and preserve left ventricular function in experimental studies. Neurohumoral activation and stunning are also dependent on the severity and duration of ischemia, which influence the magnitude of sympathetic activation and subsequent contractile dysfunction [48]. In addition, elevated catecholamines and activation of the sympathetic nervous system result in β-adrenergic desensitization with further dampening of the contractile function. CMR and clinical imaging techniques such as dobutamine stress echocardiography have been useful in understanding the extent and recovery time of myocardial stunning, and therefore its clinical significance in post-MI management [49].

#### 2.2.4. Preclinical Models and Investigated Therapeutic Strategies

Preclinical models have been pivotal in advancing our understanding of myocardial infarction (MI) and reperfusion injury, providing critical insights into the underlying mechanisms and potential therapeutic approaches. Animal models, including rodents (rats and mice), rabbits, pigs, and non-human primates, are widely used to replicate the ischemia-reperfusion (I/R) injury environment. These models enable the study of myocardial responses to ischemia and subsequent reperfusion, mimicking the clinical scenario of patients undergoing percutaneous coronary intervention (PCI) or thrombolytic therapy [50]. In parallel, in vitro models such as isolated cardiomyocyte cultures and Langendorff-perfused hearts have facilitated detailed mechanistic studies at cellular and molecular levels. Preclinical investigations of various therapeutic strategies, including ischemic preconditioning and postconditioning and pharmacologic agents that target oxidative stress, inflammation, calcium overload, and mitochondrial dysfunction, have been performed [51]. Additionally, novel approaches such as gene therapy, microRNA modulation, and stem cell-based interventions have shown promise in reducing infarct size and improving cardiac function post-reperfusion. Despite encouraging results in preclinical studies, the translation of these therapies to clinical settings remains challenging due to species differences, model limitations, and complexities of human pathophysiology [52]. Preclinical models and the investigated therapeutic strategies for myocardial infarction reperfusion injury are shown in Table 1.

## 3. Clinical Implications and Diagnosis

MI occurs as a heterogeneous disease, and the clinical management of MI involves extensive processes to identify infarction damage from reperfusion injury as well as to monitor biomarkers of reperfusion injury and assess the extent of myocardial injury through advanced imaging modalities. These elements are all of great importance in defining the magnitude of cardiac injury and therefore guide therapeutic decisions. This section will address the clinical challenges, biomarkers, and imaging protocols used to assess myocardial damage following reperfusion therapy while incorporating the most impactful studies and evidence.

After the onset of MI, one of the major clinical challenges is to differentiate infarction-related damage from reperfusion injury. Due to the prolonged lack of blood supply, infarction occurs and leads to irreversible myocardial cell death. However, on the other hand, reperfusion injury occurs when the blood flow is restored to previously ischemic tissue, resulting in further cellular damage. This distinction is important because both conditions can lead to severe long-term cardiac dysfunction, but the pathophysiology and therapeutic implications differ [53]. Reperfusion injury forms additional markers of myocardial injury, such as oxidative stress, inflammation, and calcium overload, when blood is restored. This can be difficult to detect clinically as the overlap in clinical features of both conditions can be difficult to differentiate. Reperfusion injury can further exacerbate the damage by endothelial dysfunction and microvascular injury. In general, infarction leads to myocardial necrosis, although infarcted hearts can also sustain myocardial stunning. The dual burden makes assessment of myocardial injury and recovery that much more difficult [54]. Furthermore, reperfusion can be confused with progressive infarction in some cases, thus altering the therapeutic approach. A major role of reperfusion injury in the adverse outcome of MI patients with arrhythmias, left ventricular dysfunction, and higher mortality has been established. However, infarction and reperfusion injury are usually distinguished from each other with the help of clinical markers and imaging techniques. These tools can save clinicians from misjudging the severity of acute damage or a treatment approach in patients undergoing thrombolytic therapy or PCI [55].

Monitoring of MI and determining reperfusion success depend on biomarkers. Cardiac troponins, together with lactate dehydrogenase (LDH), function as the leading biomarkers to detect infarctions and assess reperfusion injuries. These biomarkers provide essential data about the extent of myocardial tissue damage and help determine when reperfusion injury occurs. A list of the selected clinical trials investigating therapeutic strategies for myocardial infarction reperfusion injury is provided in Table 2.

### 3.1. Cardiac Troponins

Acute MI (AMI) diagnosis depends on cardiac troponins (cTnI and cTnT), which medical experts recognize as the most effective biomarkers. The detection of these proteins within blood circulation establishes myocardial damage because myocardial cells release these proteins after they become damaged. The best indicators for determining myocardial injury severity make cardiac troponins more essential than alternative cardiac markers in identifying various types of heart damage. Measuring reperfusion injury with troponins requires a more complex assessment procedure compared to the assessment of myocardial infarction [56]. Studies have shown that even after successful reperfusion, the reperfusion injury can cause troponin levels to remain elevated, despite the elevation being due to reperfusion damage. In the presence of infarction or reperfusion injury, raised troponin levels can be observed, but it is not possible to ascertain the specific cause of MI on the basis of a single biomarker. It is interesting, though, that some studies have found that, in fact, higher levels of troponin after reperfusion therapy correlate with worse outcomes, including bigger infarct size, less functional recovery, and, in some cases, an increased risk of death. Nevertheless, troponin is unable to discriminate between infarction and reperfusion injury and thus does not provide an adequate basis for understanding damage in its entirety within the context of reperfusion therapy [57].

### 3.2. Lactate Dehydrogenase (LDH)

An additional biomarker that can be assessed to determine myocardial injury is LDH, which is less specific than cardiac troponins. Myocardial cells release LDH when they are damaged, and its level rises later than that of troponin, typically within 24–48 h after injury [58]. LDH is of great value for differentiating between infarction and reperfusion injury due to its prolonged elevation after reperfusion therapy, reflecting not only the infarct-induced cell death but also the damage to the cell by oxidative stress and inflammation during reperfusion. In fact, studies have indicated that elevations of LDH (especially together with such biomarkers as cardiac troponins) can help to increase the value of the comprehensive assessment of myocardial injury. Although LDH is a nonspecific marker and is not a definitive marker for distinguishing ischemic infarction from reperfusion injury, it is, nevertheless, also a supplementary marker [59].

### 3.3. Imaging Techniques to Evaluate Myocardial Damage

Assessment of myocardial damage has been revolutionized by advanced imaging techniques, in particular, the evaluation of reperfusion injury. MRI and echocardiography are two of the most commonly used imaging modalities for clinical practice, both of which provide unique information on myocardial injury and its progression.

#### 3.3.1. Cardiac MRI

Currently, as the gold standard for the assessment of myocardial damage, cardiac MRI is able to obtain high-resolution images of the heart to quantify infarct size and identify myocardial ischemia and reperfusion injury. MRI is used to study myocardial injury using several sequences, including late gadolinium enhancement (LGE) and T2-weighted imaging. LGE MRI is able to distinguish infarct from viable tissue; it provides useful information about the exact extent and location of infarcted myocardium. T2-weighted imaging shows myocardial edema as a hallmark of reperfusion injury that can be differentiated from irreversible infarction [60]. Therefore, the promise of performing quantitative measurements of the degree of reperfusion injury based on quantified areas of the edema (indicating inflammatory or damaged tissue as a result of reperfusion) has been demonstrated using MRI. Further, MRI has the potential to provide a more precise measure of infarct size that could be relevant for predicting long-term outcomes, ventricular remodeling, and heart failure. In clinical studies in patients with reperfusion injury as assessed by MRI, patients at higher risk of experiencing adverse cardiovascular events, including arrhythmias and left ventricular dysfunction, have larger areas of reperfusion injury [61].

First-pass perfusion CMR is a pixel-by-pixel quantification of myocardial blood flow (MBF). In one multicenter study of 234 patients, first-pass perfusion MRI identified ischemic myocardium with 100% sensitivity and 93% specificity compared with conventional nuclear methods and was an independent prognostic indicator of 1-year outcomes in terms of new infarcts or death [62,63]. Deep learning (DL) facilitates MBF mapping by performing automated segmentation and one-click reporting, improving clinical utility [64]. Dynamic CT-MPI (equivalent to CMR perfusion) in diabetic patients without epicardial disease indicates a significantly lower global MBF, a greater prevalence of microvascular myocardial ischemia (~36% vs. 10% in non-diabetics), and an increased extracellular volume indicating fibrosis. CMR studies affirm that early T2D is linked to microvascular perfusion impairments and occult fibrosis, which normal LGE can overlook [65,66]. Moreover, hyperglycemia and insulin resistance increase susceptibility to reperfusion injury in both diabetic and acute stress conditions [67]. In T2D patients undergoing metformin therapy, myocardial perfusion reserve (MPR) on stress CMR is increased, and survival is better (adjusted HR, 0.24), suggesting the cardioprotective potential of metformin therapy in T2D patients. In preclinical models, metformin limits cardiomyocyte apoptosis after I/R through AMPK/PGC-1alpha signaling, which normalizes infarct size closer to that of non-diabetic animals [68,69]. Additional treatments (e.g., SGLT2 inhibitors, GLP-1 RAs) are being tested in microvascular and perfusion improvement, which can be measured by means of CMR biomarkers, including MBF and ECV, providing a translational route between metabolic modulation and imaging outcome [70,71]. Furthermore, CMR perfusion (MBF, MPR), T1/T2 mapping, and LGE can be used together to assess ischemia, edemas, fibrosis, and viability simultaneously. In combination with CT-MPI/CCTA and serum markers (e.g., HbA1c, CRP), this multiparametric approach may be able to identify high-risk diabetic patients and guide metabolic therapy as well as monitor response to treatment in clinical trials [72,73].

#### 3.3.2. Echocardiography

Echocardiography is another important tool for the evaluation of myocardial damage in acute clinical settings. Echocardiography is widely available and noninvasive and is particularly useful because it gives real-time information regarding myocardial function, although it is usually not as detailed or specific as MRI. Assessment of myocardial damage and estimation of the benefit of reperfusion treatment can be performed using echocardiographic parameters such as left ventricular ejection fraction (LVEF), regional wall motion abnormalities, and myocardial strain. Echocardiography can aid in the determination of areas of the myocardium that have been reperfused and in the recovery of myocardial function post-reperfusion in patients undergoing PCI or thrombolysis [74]. In addition, it is useful to detect reperfusion damage complications such as pericardial effusion, left ventricular thrombus, or mitral regurgitation. Nonetheless, echocardiography’s ability to differentiate between infarction and reperfusion injury is limited because it is basically functional rather than structural. However, recent developments in speckle tracking echocardiography have made it possible to evaluate myocardial strain and hence find subtle changes in myocardial function during reperfusion injury, capabilities that were not available previously. In patients with reperfusion injury, these techniques have been demonstrated to provide incremental value in predicting long-term outcomes, including heart failure and adverse remodeling [75].

**Table 2:** A list of the selected clinical trials investigating therapeutic strategies for myocardial infarction reperfusion injury.

Trial Name/ID	Phase	Intervention/Therapy	Patient Population	Targeted Mechanism	Outcome/Findings	Reference
CIRCUS (NCT01502774)	Phase III	Cyclosporine A	STEMI patients undergoing PCI	Mitochondrial permeability transition pore inhibition	No significant reduction in infarct size or MACE	[59]
MITOCARE (NCT01374321)	Phase II	TRO40303 (Mitochondrial protector)	STEMI patients	Mitochondrial membrane stabilization	No significant cardioprotective effect observed	[60]
DANAMI-3-iPOST (NCT01435408)	Phase III	Ischemic postconditioning (iPOST)	STEMI patients undergoing PCI	Reduction of reperfusion injury via brief intermittent reperfusion	No significant benefit in clinical outcomes	[61]
ELIXIR (NCT00091637)	Phase III	Exenatide (GLP-1 agonist)	STEMI patients	Cardiomyocyte protection, metabolic modulation	Modest reduction in infarct size	[74]
NOMI trial	Phase II	Nitric oxide inhalation	STEMI patients during PCI	Microvascular protection, vasodilation	Improved myocardial perfusion but mixed results on infarct size	[75]

## 4. Current and Emerging Therapeutic Strategies

### 4.1. Pharmacological Interventions

**Figure 4:**
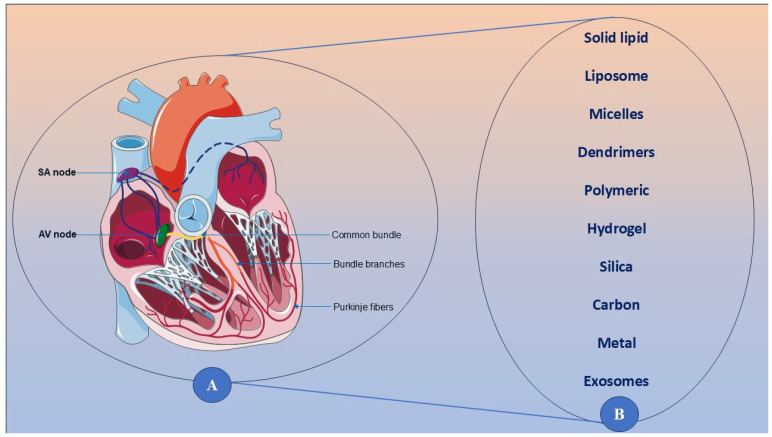
(**A**) Normal anatomy of the heart showing the key structural components, including the atria, ventricles, major blood vessels (aorta, pulmonary arteries and veins), coronary arteries, and valves. This panel provides a baseline anatomical reference for understanding the cardiac function and pathology. (**B**) Schematic diagram illustrating therapeutic strategies utilizing nanomaterials for myocardial infarction reperfusion injury.

Despite the extensive research on pharmacological strategies to reduce myocardial damage in the setting of MI, reperfusion injury remains a major challenge in the management of MI. Therapeutic agents from several classes, including antioxidants and ROS scavengers, calcium channel blockers and mitochondrial stabilizers, anti-inflammatory agents, and ischemic conditioning strategies, have been explored to protect against ischemia-reperfusion injury (IRI). Some of these interventions have shown promise in preclinical and early-phase clinical studies, but their ability to translate to routine clinical use is under active investigation [76]. Normal cardiac anatomy and nanomaterial-based therapeutic strategies for myocardial infarction reperfusion injury are presented in Figure 4.

#### 4.1.1. Antioxidants and ROS Scavengers

Reperfusion injury involves oxidative stress, which is generated from an excessive generation of ROS following sudden blood flow restoration, including lipid peroxidation, protein modification, mitochondrial dysfunction, and DNA damage. Several pharmacological strategies have been tested that target oxidative stress. Superoxide dismutase (SOD) mimetics, including tempol, have been studied for their ability to reduce ROS levels and mitigate oxidative damage among tissues [77]. In addition, N-acetylcysteine (NAC), a glutathione precursor, has shown potential to restore intracellular antioxidant reserves and reduce oxidative stress. Other strong ROS scavengers, such as edaravone, have been shown to have cardioprotective effects by inhibiting lipid peroxidation and reducing oxidative injury. In addition, melatonin, with its antioxidant and anti-inflammatory activity, inhibits infarct size and maintains mitochondrial function in experimental models of IRI. Despite these promising results, clinical trials attempting to determine if antioxidants can reduce myocardial injury through reperfusion have reported a mixture of results. The complex interaction between ROS and the cellular signaling pathways involved during reperfusion is believed to contribute to their increased variability. The randomized, double-blind NACIAM trial (112 STEMI patients) showed that high-dose NAC plus low-dose nitroglycerin decreased infarct size (11.0% vs. 16.5%; *p* = 0.02) and almost doubled myocardial salvage on cardiac MRI (60% vs. 27%; *p* < 0.01) [78]. A smaller trial also found enhanced TIMI grade 3 flow (94% vs 80%; *p* = 0.03) and reduced peak hs-TnT with NAC [79]. These trials present premature clinical evidence in support of antioxidant therapy, but further, larger studies are required to relate surrogate markers to definitive long-term outcomes. Additional support for these data comes from a meta-analysis of 28 RCTs (n = 2174) that demonstrated NAC tendencies to lower cardiac troponin release and postoperative atrial fibrillation, and seven of nine ROS-marking trials associated decreased oxidative stress with improved clinical outcomes [80].

#### 4.1.2. Calcium Channel Blockers and Mitochondrial Stabilizers

Reperfusion injury is a major source of calcium overload in the heart, especially during the opening of the mPTP and cardiomyocyte hyper-contracture, which potentially results in necrotic cell death. To attenuate these effects, pharmacological agents targeting calcium homeostasis and the stability of mitochondria have been investigated. Such calcium channel blockers as verapamil and diltiazem have been investigated as potential means for decreasing calcium influx and/or limiting myocardial damage [12]. In experimental models of IRI, these agents have shown efficacy, but there has been insufficient evidence of benefits in clinical settings. Because of its ability to inhibit mPTP opening and thus preserve mitochondrial integrity and decrease apoptosis, mitochondrial stabilizers, such as cyclosporine A, are potential cardioprotective agents. In a 2019 preclinical study, a mitochondria-targeted CsA delivery system (CsA@PLGA-PEG-SS31) was used in rat IRI models, where it demonstrated substantial preservation of mitochondrial integrity, reactive oxygen species suppression, decreased apoptosis, and reduced infarct area, confirming the mechanistic potential of CsA [81]. Cyclosporine A has been evaluated in clinical trials that have reported variable results, and some studies have reported a reduction in infarct size, though no improvement in clinical outcomes has been observed with others. Other mitochondria-targeted agents, including sanglifehrin A and elamipretide, have also been explored, with encouraging preclinical results, but their efficacy in human phase trials remains unproven. Clinical testing has been performed on mitochondrial stabilizers, including cyclosporine A (CsA). In a small pilot study (58 STEMI patients), a single IV dose of CsA at reperfusion minimized peak CK release (*p* = 0.04) and infarct mass on MRI (37 g vs. 46 g; *p* = 0.04) [82]. The multicenter CIRCUS trial (n = 970 antecedent STEMI patients) tested intravenous cyclosporine A given just before PCI. Despite encouraging results in small pilot trials, CIRCUS finally showed that at 1 year, cyclosporine failed to lower heart failure rates or mortality or adverse LV remodeling, and echocardiographic measurements showed no improvement in systolic or diastolic function compared with a placebo [83]. The randomized, multicenter CYCLE trial involved 410 STEMI patients receiving a single IV dose of CsA (2.5 mg/kg) just before primary PCI, with the outcome measured in terms of ST-segment resolution, high-sensitivity troponin T (hs-cTnT), left ventricular (LV) function, and 6-month event rates. The outcome did not significantly favor the 60 min achievement of 60–70% ST-segment resolution (52% CsA vs. 49% control; *p* = 0.55), and there were no differences in median day 4 hs-cTnT (2160 vs. 2068 ng/L; *p* = 0.85), LV ejection fraction, or clinical outcomes, including mortality [84]. These results are in line with similar findings in the CIRCUS trial, which also did not find an infarct size reduction, adverse LV remodeling, or major cardiac event reduction [85].

#### 4.1.3. Anti-Inflammatory Agents

Inflammation is a key mediator of reperfusion injury, with neutrophil infiltration, pro-inflammatory cytokine release, and endothelial dysfunction contributing to myocardial damage. Targeting the inflammatory cascade through pharmacological interventions has been an area of active research. Interleukin-1 (IL-1) inhibitors, such as anakinra and canakinumab, have demonstrated potential in reducing myocardial inflammation and limiting infarct size. These agents work by blocking IL-1 signaling, which plays a central role in inflammatory cell recruitment and tissue injury following reperfusion [86]. Corticosteroids, although effective in reducing systemic inflammation, have shown limited benefit in myocardial reperfusion injury due to concerns over immunosuppression and adverse metabolic effects. Nonetheless, targeted inhibition of specific inflammatory pathways, such as neutrophil elastase inhibitors and complement cascade inhibitors, has demonstrated cardioprotective effects in preclinical models. Other novel anti-inflammatory agents, including colchicine and monoclonal antibodies targeting tumor necrosis factor alpha (TNF-α), have been evaluated for their ability to reduce myocardial inflammation and improve cardiac function post-reperfusion. While some studies suggest a potential benefit, further clinical trials are needed to establish their efficacy in routine clinical practice [87]. Moreover, glucagon-like peptide-1 (GLP-1) receptor agonists have been identified as promising compounds with both anti-inflammatory and cardiometabolic effects. Current evidence published by MDPI indicates that GLP-1 and GLP-1R agonists stimulate myocardial microvascular perfusion through the upregulation of endothelial nitric oxide synthase (eNOS) and vascular endothelial growth factor (VEGF) and diminish the vasoconstrictor endothelin-1 (ET-1), thereby protecting the NO bioavailability and augmenting microvascular dilation. They also enhance acetylcholine-induced vasodilation, reduce oxidative stress and vascular inflammation, polarize macrophages towards anti-inflammatory M2 rather than pro-inflammatory M1 phenotype, and promote angiogenesis through HIF-1 alpha/VEGF signaling—collectively enhancing coronary microcirculation. In particular, agents including exenatide and liraglutide were found to activate the AMPK/PI3K/Akt/eNOS signaling pathways in endothelial cells, stimulate NO production, and ameliorate microvascular and systolic dysfunction in animal and clinical models. Semaglutide profoundly enhanced myocardial perfusion and left ventricular systolic performance in swine models of coronary disease, partly because of AMPK-eNOS activation, and also decreased fibrosis and apoptosis. Although these vasoprotective, perfusion-enhancing effects are impressive, additional clinical studies are required to strengthen the efficacy and everyday practicability of GLP-1R agonists in ischemia-reperfusion injury and myocardial perfusion augmentation [88].

#### 4.1.4. Remote Ischemic Conditioning and Preconditioning Strategies

Ischemic conditioning strategies, including remote ischemic conditioning (RIC) and ischemic preconditioning (IPC), have been explored as non-invasive approaches to mitigate reperfusion injury. RIC involves the application of brief cycles of ischemia and reperfusion to a remote organ or limb before, during, or after myocardial ischemia, triggering endogenous protective mechanisms that reduce myocardial damage. RIC reliably decreases infarct size in rodent models in preclinical settings, with many studies demonstrating that this preconditioning also positively regulates various pathways (e.g., miR-1/BDNF or JAK-STAT signaling), and others showing that mitochondrial and electrophysiological parameters are preserved [89]. Several clinical trials have shown the cardioprotective effects of RIC, and there is now evidence that RIC may reduce infarct size and improve clinical outcomes in patients undergoing PCI. Meta-analyses of approximately 23 randomized trials (~2100 STEMI and 2000 elective PCI patients) suggest that RIC has a modest infarct size-reducing effect of ca. 2–3% of the left ventricular volume (MD, −2.43%; 95% CI, from −4.37 to −0.48), decreases the release of cardiac biomarkers, and has a small positive effect on early post-STEMI LVEF [90]. In some small trials (e.g., OR for mortality, ~0.27; MACCE, ~0.45), mortality and major adverse cardiovascular and cerebrovascular event (MACCE) rates were also lowered [91]. IPC, whereby short periods of ischemia are elicited prior to a prolonged ischemic event, has been shown to activate protective cellular pathways, including protein kinase C, adenosine receptors, and mitochondrial ATP-sensitive potassium channels [92]. Despite its strong protective effect in experimental models, IPC has not been clinically applicable owing to challenges associated with timing and feasibility. Mechanical IPC has also been accepted as an alternative to pharmacological preconditioning using agents such as adenosine receptor agonists and nitric oxide donors. These approaches comprise strategies that seek to activate endogenous cardioprotective pathways, which mimic the protective effects of IPC. Although preclinical studies have demonstrated promising results, clinical trials investigating the efficacy of pharmacological preconditioning strategies failed to reach similar conclusions and deserve further exploration [93].

### 4.2. Non-Pharmacological Strategies

Owing to its adverse effects and poor efficacy, the management of myocardial ischemia-reperfusion injury has evolved to include a number of non-pharmacological strategies to limit damage and promote myocardial repair. These approaches aim to affect cellular and molecular pathways of ischemic injury beyond what is possible with conventional pharmacotherapy. Hypothermia and metabolic modulation, gene therapy and regenerative approaches, as well as stem cell therapies are among the most promising interventions that have all been involved in the protection and repair of the myocardium.

#### 4.2.1. Hypothermia and Metabolic Modulation

Hypothermia has now been shown to be a promising intervention for reducing ischemia-reperfusion injury by lowering myocardial oxygen demand and inhibiting the proinflammatory and dysregulated biochemical cascades due to oxidative stress and inflammation. Preservation of mitochondrial integrity, prevention of calcium overload, and reduction of the inflammatory response have all been demonstrated to occur in the presence of therapeutic hypothermia and are known contributors to myocardial injury during reperfusion. Studies, both experimental and clinical, have shown mild-to-moderate hypothermia (32–34 degrees Celsius) decreases infarct size and improves cardiac function by inhibiting apoptotic signaling and stabilizing cellular membranes [94]. Hypothermia regulates important metabolic pathways at the molecular level by reducing ATP consumption and the generation of ROS. The downregulation of pro-apoptotic proteins, including Bax and caspase-3, and the induction of anti-apoptotic molecules such as Bcl-2 have been observed to contribute to this protective effect. Hypothermia has also been demonstrated to suppress neutrophil activation and, as a result, decrease cytokine-driven endothelial dysfunction and microvascular obstruction. Some research indicates that controlled cooling just before reperfusion, commonly referred to as ischemic postconditioning, additionally elevates cardioprotective properties by preventing oxidative stress-triggered lipid peroxidation and conserving mitochondrial function [95]. In addition to hypothermia, metabolic modulation strategies optimize energy metabolism in ischemic cardiomyocytes. To shift the myocardial metabolism from fatty acid oxidation to glucose oxidation, thereby improving energy efficiency and decreasing ROS production, metabolic modulators such as trimetazidine and ranolazine have been studied as interventions targeting the glucose and fatty acid metabolism. This metabolic shift is the second line of defense promoting ATP generation and reducing oxidative stress to further protect from ischemia-reperfusion injury. There is preclinical and clinical evidence supporting the preservation of cardiac function and reduction in infarct size, attributes of metabolic modulation as a valuable addition to hypothermia in cardioprotective strategies [96].

#### 4.2.2. Gene Therapy and Regenerative Approaches

Myocardial repair after ischemia-reperfusion injury has been an area of continued focus on which gene therapy may provide a potential therapeutic strategy. Gene therapy introduces genetic material into cardiac cells in order to modify the main pathways that control myocardial survival, angiogenesis, and tissue regeneration. Several studies in preclinical and clinical settings have been performed to establish the utility of gene therapy to improve myocardial repair by activation of endothelial nitric oxide synthase (eNOS), angiogenesis mediated by vascular endothelial growth factor (VEGF), and inhibition of apoptosis [97]. VEGF is among the most studied gene therapy approaches to enhance myocardial perfusion and hence improve myocardial function through the delivery of VEGF to stimulate neovascularization. VEGF gene therapy in vascular models of myocardial infarction augments capillary density, restores endothelial function, and reduces infarct size, as demonstrated in the experimental models. Additionally, other angiogenic factors that have been studied for their ability to facilitate collateral vessel formation and improve myocardial tissue oxygenation include fibroblast growth factor (FGF) and hepatocyte growth factor (HGF) [98].

The strategies targeting the anti-apoptotic pathways other than angiogenesis have demonstrated promising results in ischemic injury reduction using gene therapy. Genes that have anti-apoptotic functions, such as Bcl-2 and Akt, have been shown to protect cardiomyocytes from apoptosis along with inhibition of mPTP opening and decreased caspase activation. The gene transfer of heat shock proteins (HSPs) also increases cellular resistance to oxidative stress and helps avoid myocardial dysfunction with reperfusion [99]. MicroRNA (miRNA)-based therapies are another innovative form of gene therapy. MicroRNAs have also been implicated in different aspects of cardiac remodeling and repair and post-transcriptionally regulate gene expression. Such examples include certain miRNAs, for instance, miR-21 and miR-126 are favorable miRNAs that enhance angiogenesis, suppress proinflammatory signaling pathways and are protective to the myocardium. Therefore, directed gene therapy aimed at modulating these miRNAs has the potential to increase myocardial recovery and decrease adverse remodeling following ischemia-reperfusion injury [100].

#### 4.2.3. Stem Cell-Based Interventions for Myocardial Repair

Based on the potential use of stem cell therapy to repair damaged myocardium and restore cardiac function after ischemia-reperfusion injury, stem cell therapy is one of the most promising regenerative strategies. The potential of stem cells to differentiate into multiple cardiac cell types, secretion of paracrine factors promoting repair of the tissue, and contribution to inflammatory responses modulating myocardial recovery are hallmarks of changes that occur with stem cell therapy. Mesenchymal stem cells (MSCs), cardiac progenitor cells (CPCs), and induced pluripotent stem cells (iPSCs) have been molecularly studied as being the most promising novel myocardial stem cells. Mesenchymal stem cells have recently been identified as being capable of releasing proangiogenic, anti-apoptotic factors and have immunomodulatory properties [101]. The release of growth factors such as VEGF, HGF, and IGF-1, as well as evidence for infarct size decreases, improvement of left ventricular function, and neovascularization from preclinical and clinical studies, demonstrates that MSCs can reduce infarct size, increase left ventricular function, and promote neovascularization. Furthermore, MSCs have been reported to participate in the control of fibrosis and attenuation of maladaptive cardiac remodeling, and, thus, these cells are good candidates for myocardial repair [102].

Cardiac progenitor cells (CPCs) are some of the other promising cell types for myocardial regeneration. The cells with the most potential that have been derived from the heart itself are these, which already show the inborn potential to develop into cardiomyocytes, endothelial cells, and smooth muscle cells. CPC transplantation has been reported to improve cardiac function and reduce infarct size in clinical trials, with evidence that CPCs exert most of their beneficial effects via paracrine signaling rather than direct cell differentiation. However, the potential of induced pluripotent stem cells (iPSCs) to provide an organic, patient-specific source of cardiomyocytes for myocardial repair is an exciting avenue and has experimentally demonstrated the ability of iPSC-derived cardiomyocytes to integrate into host myocardium, restore electrical conduction, and improve the contractile function in animal models of myocardial infarction [103]. Despite these advances, various challenges, including immune rejection, tumorigenicity, and the need for optimized differentiation protocols, remain to be overcome before the use of iPSC-based therapies becomes widespread in clinical practice. A novel approach for myocardial repair based on exosome-based stem cell therapy has also been described. Stem cell exosomes, small extracellular vesicles that are secreted by stem cells, allow for intercellular communication mediated by proteins, lipids, and miRNAs and are also involved in tissue regeneration. Moreover, studies have found exosome therapy could reduce apoptosis and enhance angiogenesis and improve cardiac function following ischemia-reperfusion injury, and thus exosome therapy has the potential to be a cell-free regenerative strategy [104].

### 4.3. Advanced Nanomedicine Approaches

In the context of MI and its associated reperfusion injury, nanomedicine has emerged as a cutting-edge therapeutic platform offering targeted and multifunctional solutions. Traditional reperfusion therapies, while critical for restoring the blood flow, often exacerbate myocardial injury through oxidative stress, inflammation, and limited cardiomyocyte survival. Advanced nanomedicine approaches aim to overcome these limitations by employing engineered nanoparticles, liposomes, hydrogels, and biomimetic systems for the precise delivery of drugs, genes, antioxidants, and growth factors directly to the ischemic myocardium [105]. These nanosystems offer enhanced bioavailability, prolonged circulation time, and site-specific accumulation, thereby reducing off-target effects and improving therapeutic efficacy. Moreover, nanomaterials are being designed to actively modulate key pathological processes such as cardiomyocyte apoptosis, inflammation resolution, neovascularization, and extracellular matrix remodeling. While many of these strategies are currently at the preclinical stage, their integration into MI treatment paradigms holds the potential to revolutionize cardiac repair and reduce the progression to heart failure following reperfusion injury [106].

## 5. Network Pharmacology Approaches in MIRI

Network pharmacology has the potential to influence the drug development process at multiple stages, including target identification, lead discovery and optimization, mode of action, preclinical efficacy, and safety evaluation [107]. In turn, this could make it easier to systematically describe drug targets, which would help lower the usually high rate of project failure that comes with discovery efforts. Based on the known and predicted protein–protein interaction data for many organisms, the STRING database shows both physical interactions and functional associations, along with confidence scores that show how reliable the data are. Cytoscape (version 3.8.0), on the other hand, is built for analyzing and visualizing massive networks, and it gives users a lot more leeway when it comes to adding new data and displaying it on top of the existing networks. In Cytoscape, networks are graphs with nodes (genes and proteins) and edges (reactions). In this experimental investigation, a degree indicates the number of a node’s immediate neighbors. The more directly related nodes a node has, the greater its influence. A node’s betweenness centrality is determined by the shortest path that can be taken between any two other nodes via that node. Closeness centrality is defined as the reciprocal of the average shortest path distance between a node and all other nodes in the network; the greater the value, the greater the centrality of the node, indicating that the signal is transmitted from one node to other nodes more rapidly. The network’s nodes represent the target or active component, while the edges denote how the two components interact with one another. Each of the nodes is either a protein or an active component. Biological molecules communicate with each other through the edge between nodes. The highest degree indicates that the targeted genes have a significant correlation with each other; as a result, all these genes may be potential critical targets.

**Figure 5:**
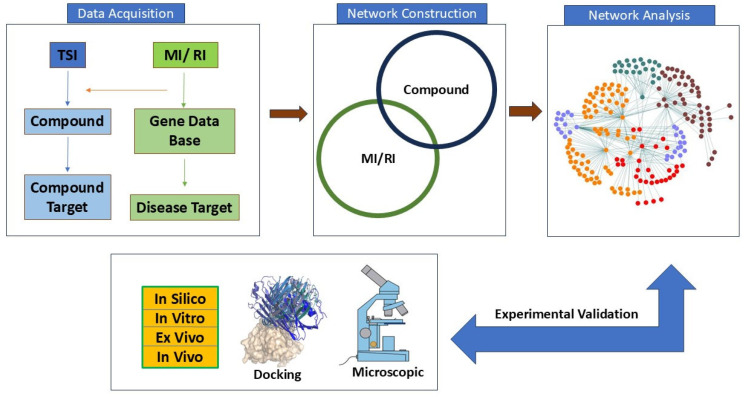
The flowchart of network pharmacology in MIRI. The illustration shows a step-by-step drug discovery workflow with a network pharmacology approach. It starts with data acquisition, in which compound data are obtained based on the traditional system of information (TSI) and modern/recent information (MI/RI), and then compound and disease targets are identified through gene databases. These targets are subsequently incorporated into the construction of the network to determine the shared targets between the compounds and diseases, which is presented in a Venn diagram fashion. The resulting network is thoroughly analyzed so as to identify important molecular interactions and pathways. Lastly, the in silico predicted targets and interactions undergo experimental verification to ascertain their therapeutic context.

Genes that have many connections with other genes are termed “hub genes” in gene networks. These usually play an essential role in gene regulation and biological processes. A high-degree node, or hub, is the core component of any kind of network. When compared to other nodes in the network, hubs have an extremely high link density. If the number of links between the hubs is the same as would be predicted by chance, this network is referred to as a neutral network. When hubs exhibit a tendency to connect with other hubs while avoiding links with nodes of a low degree, the network can be described as an assortative network. Because hubs form a core group that is more resilient to hub removal, this network is comparatively resistant to attacks. When hubs refrain from connecting to one another while establishing links with nodes of a lower degree, the network in question is commonly referred to as a disassortative Network. The flowchart of network pharmacology in MIRI is outlined in Figure 5.

Acute MIRI evokes significant changes in lipid metabolism, fibrinolysis, and extracellular matrix remodeling—processes in which the main genes, such as *APOB*, *SERPINE1*, and *TIMP1*, are involved. *APOB*, which is critical in the development of LDL, could contribute to reperfusion injury-related oxidative stress and endothelial dysfunction because of the promotion of lipid accumulation and oxidation throughout the reperfusion, which worsens microvascular block and infarct growth. The central plasminogen activator inhibitor, *SERPINE1* (PAI-1), has a dual role in this respect: its early increase helps to limit hemorrhagic complications, but its persistent elevation compromises fibrinolysis, contributing to microthrombi and microvascular plugging, which worsen reperfusion outcomes. Conversely, *TIMP1* regulates the activity of matrix metalloproteinases to maintain the integrity of the extracellular matrix in the process of reperfusion. In experimental models, *TIMP1* deficiency results in increased neutrophil infiltration, matrix degradation, and impaired tissue repair following I/R. Therefore, any therapeutic approaches resulting in an increased stability of *TIMP1* offer a promising avenue of work to regulate inflammation, ECM preservation, and microvascular integrity in MIRI [108]. Network pharmacology has been effective in revealing these targets on a systems level. Using a combination of bioinformatic databases (e.g., GeneCards, OMIM) and compound target prediction algorithms (e.g., PharmMapper) in conjunction with protein–protein interaction networks, scientists have repeatedly highlighted *APOB*, *SERPINE1*, and *TIMP1* as part of the 90–150 hub genes involved in the pathophysiology of MI/RI. Functional enrichment analyses also attribute these genes to lipid metabolism, fibrinolytic balance, ECM homeostasis, inflammation, oxidative stress, and apoptosis, which are the fundamental aspects of reperfusion pathogenesis. These targets were experimentally verified by case studies (e.g., total salvianolic acid, Shuxin decoction) showing enhanced cardiac results by regulating these pathways. Therefore, network pharmacology identifies *TIMP1* as a highly favorable biomarker target, highlighting its reparative role in ECM homeostasis and neutrophil-mediated inflammation, whereas *APOB* and *SERPINE1* crosslink metabolic and hemostatic pathways in reperfusion injury. These combination therapies form a strong basis for multi-targeting therapeutics that can be used to modulate lipid metabolism, proteolysis, and tissue integrity in acute myocardial reperfusion [26]. Together, *TIMP1*, *APOB*, and *SERPINE1* are novel and therapeutically tractable targets in the setting of MIRI, discovered via a network pharmacology strategy that facilitates the identification of systems-level regulators that would be missed in a single target-focused analysis. *TIMP1* and *SERPINE1* are actionable as therapeutic targets: they can be inhibited (with small molecules or biologics) to limit post-ischemic inflammation, ECM degradation, fibrosis, and microvascular damage; *APOB*-targeted therapies (*PCSK9* inhibitors or antisense oligos) could modulate lipid-mediated oxidative stress or membrane integrity in reperfused myocardium; and all three are omics-detectable, enabling biomarker-driven stratification [109]. Network pharmacology also provides solutions to the common pitfalls of trials, especially patient heterogeneity [110]. These methods can be used to determine responders through target expression or regulatory patterns by mapping multi-omics (genomic, transcriptomic, and proteomic) disease modules and overlaying drug–target networks [111]. As an example, the new POINT platform incorporates multi-omics networks through random-walk and knowledge graph-based approaches, which enhances target prediction and allows stratified trial design on the basis of individual molecular profiling [112]. In this manner, *SERPINE1* inhibitors, or *TIMP1*-modulating drugs, may be evaluated in patients with specific transcriptomic signatures indicating activation of ECM/inflammatory modules, which would allow greater statistical power and lower rates of trial failure.

**Figure 6:**
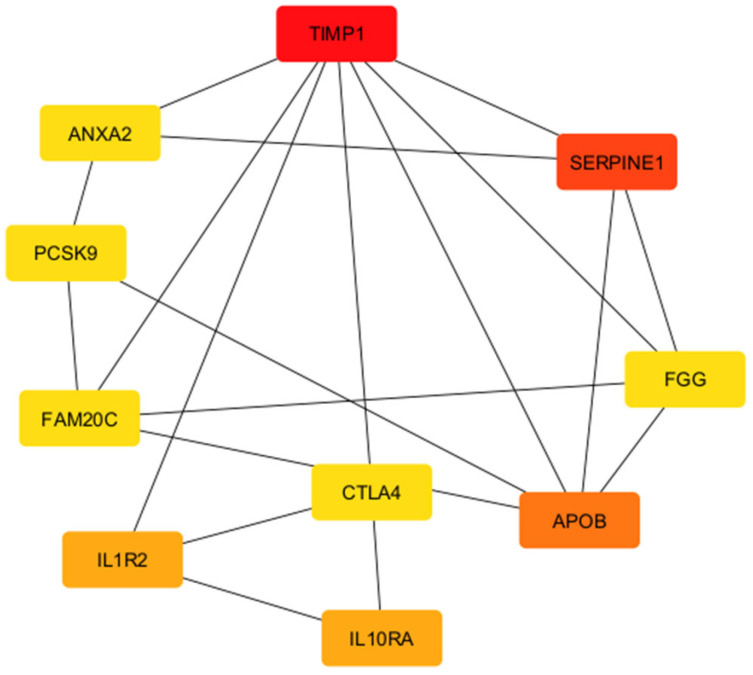
Interaction network between the hub genes and the other key genes in MIRI.

Table 3 depicts *TIMP1* as a central hub target in the generated network, which highlights the essentiality of this gene among the genes typically related to myocardial infarction (MI). A multi-database approach was used to derive these targets, including four key sources: GeneCards, where genes are prioritized by their MI relevance scores (higher scores correspond to stronger associations); DrugBank, which annotates the approved drugs used to treat MI; PharmGKB, which focuses on genes involved in drug metabolism and response; and PubChem, which helps provide bioassay data supporting an association with MI. According to this thorough strategy, the top three most significant targets out of the top 10 candidates were *TIMP1*, *SERPINE1*, and *APOB*, as illustrated in Table 3. This was the prioritization criterion used in the selection of therapeutically actionable targets. Moreover, the interaction network of hub genes with their interacting partners is shown in Figure 6, which further supports the centrality of *TIMP1*. Remarkably, *TIMP1* has become a mechanistically distinct and druggable node in MIRI, which is corroborated by recent in vivo experiments. In animals studying MIRI, co-administration of *TIMP1* with tissue kallikrein-1 (TK1) was found to considerably lessen oxidative stress and collagen deposition, decrease infarct size, induce angiogenesis, and maintain cardiac function [113]. These observations point to its therapeutic value, particularly in antifibrotic therapy and gene modulation approaches. On the other hand, *APOB*, even though not new in the ischemia setting, has an important indirect role in facilitating atherosclerosis—a key contributor to MIRI [114]. *APOB* has long been a target in lipid-lowering therapy, and agents such as PCSK9 inhibitors have proven successful in reducing LDL particles and mitigating the occurrence of MI. Overall, *TIMP1* is an attractive new target in MIRI because it regulates fibrosis and remodeling, whereas *APOB* remains clinically relevant as it mediates the progression of atherosclerosis and has already shown pharmacological tractability. Their classification as hub genes is thus biologically plausible and clinically relevant.

### 5.1. Methodology

Four databases (accessed on 12 April 2025) were initially utilized to identify genes related to myocardial infarction (MI): GeneCards (https://www.genecards.org/) to extract genes and their relevance scores to MI (with higher scores indicating a stronger relationship), DrugBank (https://www.drugbank.ca/) to extract the approved drugs used to treat MI and their targets, PharmGKB (https://www.pharmgkb.org/) to extract the genes involved in drug response and metabolism in the context of MI treatments, and PubChem (https://pubchem.ncbi.nlm.nih.gov). Then, a gene interaction network was built based on pharmacology using the Cytoscape software (version 3.8.0), which gave a visual impression of the connectivity of the genes. Enrichment analysis, such as KEGG (Kyoto Encyclopedia of Genes and Genomes) pathway analysis, was conducted to illustrate the underlying molecular mechanisms using the “clusterProfiler” package in the R software (version 3.4.0). Based on this, a protein–protein interaction (PPI) network was constructed through the STRING database (https://string-db.org/) (accessed on 13 April 2025) with a moderate confidence of 0.400, containing only the interactions that had been demonstrated by high-quality combined evidence. The resulting network was loaded into Cytoscape to further examine important subnetworks. There were two analytical approaches used to determine core targets. To build a core subnetwork, Cytoscape with the CytoNCA plugin was employed to measure network centrality parameters such as betweenness (control points in the network), closeness (fast communication potential), degree (connectivity), eigenvector, and LAC, and only genes with a higher score than the median were kept. The same filtering was completed again to narrow down the final critical subnetwork. Second, Cytoscape with the CytoHubba plugin was utilized, where the Degree and MCC (Maximal Clique Centrality) scoring algorithms were used to find and rank the top 10 hub genes without the intermediate node filtering. Such a combined strategy secured the identification of the most influential and biologically meaningful targets in the MI-related gene network in a rigorous manner.

#### 5.1.1. Molecular Target

In network pharmacology, one of the most important steps is locating potential pharmacological targets. In recent years, network pharmacology has had an influence on various aspects of life sciences, including drug mechanisms and development as well as new target discovery. The efficient identification of drug targets is one of the major challenges for drug discovery and drug development. We show how integrative network pharmacology may find drug targets and candidate genes using public database data. Identification of drug targets is one of the main tasks in drug discovery. The growth of systems biology and the rise of network pharmacology have made it possible to combine different types of data and information in drug studies. A crucial molecule in a signaling pathway that is unique to a medical condition is what we mean when we talk about a drug target. Identifying a drug’s target is an essential first step in the discovery and development of novel drugs. Finding new drug targets through network pharmacology analysis could be a useful and creative way to move forward in this direction [115].

#### 5.1.2. Gene Enrichment Analysis

Gene set enrichment analysis, which is also called functional enrichment analysis or pathway enrichment analysis, is a computational technique in which the classes of genes or proteins that are overrepresented in a large set of genes or proteins or may have an association with different phenotypes are identified. It is necessary to check if a specific group of genes is known to be involved in a specific function or pathway [116,117]. For this analysis, all query genes were first converted to ENSEMBL gene IDs or STRING-db protein IDs. For the 20 most studied species, we also manually collected a large number of pathways from various public databases. See example outputs in the Table 4.

**Figure 7:**
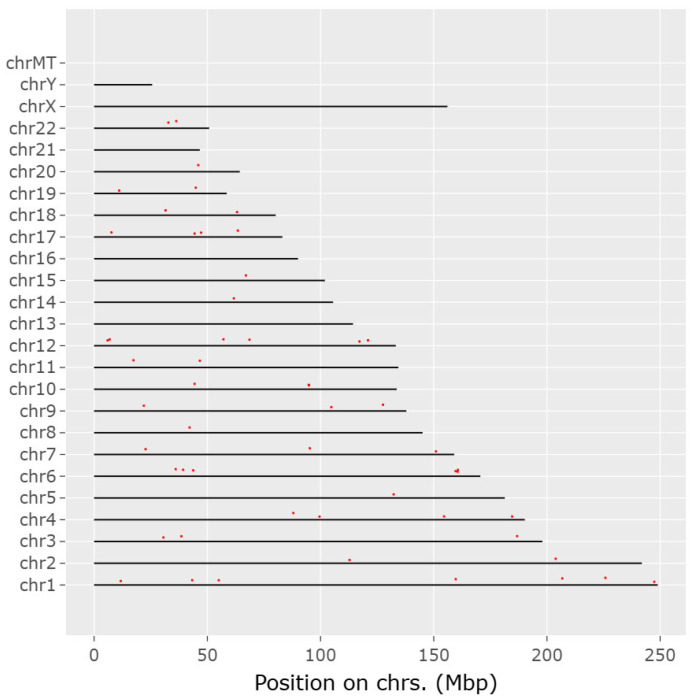
Genomic regions of statistical enrichment highlighting MIRI-associated genes. In the figure, the genes are represented by red dots. The Black lines indicate the regions where these genes are statistically enriched compared to the density of genes in the background.

In Table 4, FDR is calculated based on the nominal *p*-value from the hypergeometric test. Fold enrichment is defined as the percentage of genes in the list belonging to a pathway, divided by the corresponding percentage in the background. FDR denotes how likely the enrichment is by chance. Due to increased statistical power, large pathways tend to have smaller FDRs. As a measure of effect size, fold enrichment indicates how drastically genes of a certain pathway are overrepresented. This is an important metric, even though it is often ignored. The genomic regions of statistical enrichment highlighting MIRI-associated genes are illustrated in Figure 7.

#### 5.1.3. KEGG Pathway Analysis

Kyoto Encyclopedia of Genes and Genomes, a comprehensive bioinformatics database, is used to provide information related to genes, genomes, pathways, diseases, and drugs (see Figure 6) [118,119].

**Figure 8:**
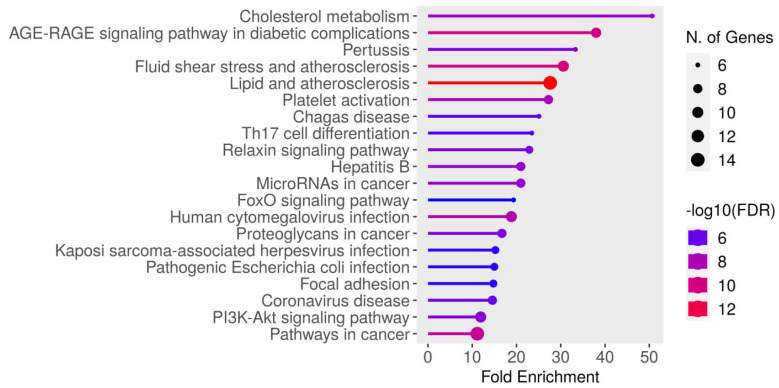
KEGG pathway analysis highlighting fold enrichment, gene count, and statistical significance (−log_10_[FDR]) in MIRI.

In this analysis (Figure 8), −log_10_(FDR) means a transformed false discovery rate (FDR) value, often used to visualize and compare the significance of GO terms. A lower –log_10_(FDR) value shows a more significant enrichment. FDR is a measure of the proportion of false positives within a set of statistically significant results. On the other hand, fold enrichment measures how much more frequently a specific group of genes (e.g., those involved in a particular biological process) is found in a gene list compared to their overall prevalence in the genome. Here, the larger the size, the more genes there are.

## 6. Challenges and Future Directions

Despite substantial progress in elucidating the mechanisms and potential therapies for myocardial infarction reperfusion injury (MIRI), several challenges continue to impede clinical translation. One of the foremost issues is the complex and multifactorial nature of MIRI, which involves an intricate interplay of oxidative stress, calcium overload, inflammation, mitochondrial dysfunction, and cell death pathways. This complexity necessitates multi-target therapeutic strategies, yet most current interventions focus on isolated pathways, limiting their effectiveness [120]. Additionally, the variability in patient populations, comorbidities (such as diabetes and hypertension), and genetic factors contribute to inconsistent clinical outcomes and limit the generalizability of preclinical findings. Another significant barrier is the gap between successful preclinical studies and disappointing results in clinical trials. Animal models often fail to fully replicate the human condition, particularly with respect to coronary anatomy, comorbid disease states, and myocardial remodeling dynamics. In addition, the existing imaging and biomarkers are not sufficiently specific to distinguish reperfusion injury from the underlying infarct, making the assessment of treatment efficacy difficult [121].

Future directions should concentrate on developing integrated, multi-target therapeutic regimens directed towards decreasing oxidative stress, inflammation, and mitochondrial injury. The incorporation of omics-based precision medicine approaches—including genomics, proteomics, and metabolomics—may identify novel molecular targets and stratify patients based on individualized risk profiles. Furthermore, some emerging technologies such as nanomedicine, gene-editing tools (e.g., CRISPR/Cas9), and bioengineered stem cell therapies promise to overcome the existing limitations and improve cardiac repair [122]. AI and ML have started revolutionizing target identification in MIRI research, screening high-dimensional -omics data, and estimating key nodes in pathological networks. As an illustration, machine learning systems trained on genomics, proteomics, and metabolomics data are revealing ferroptosis modulators (such as GPX4 modulators) as possible multi-target drug candidates [123]. Further, explainable AI (XAI) tools are used to determine the biological relevance of the predicted targets to enhance the translational potential. In the diagnostic arena, ML-based biomarker panels, especially circulating microRNAs, are reaching high sensitivity and specificity in detecting MI (e.g., miR-499 and miR-133a have reached ~0.88 sensitivity / 0.87 specificity) [124]. In addition, early prediction of reperfusion injury is being enhanced by AI-enhanced cardiac troponin assays and ensemble techniques (e.g., random survival forests), but cohort-to-cohort variation is an issue [125,126]. Regulatory frameworks are, however, not kept up to date, especially when it comes to the validation of multi-analyte biomarker panels and AI-based diagnostics. There is the problem of demonstrating clinical and cost effectiveness, acceptable standards of interpretability, and model generalizability across multiple populations.

## 7. Conclusions

Myocardial infarction reperfusion injury remains a major clinical challenge that undermines the success of reperfusion therapies and contributes to adverse cardiac remodeling and poor patient outcomes. The pathophysiology of MIRI is characterized by a cascade of deleterious events, including oxidative stress, calcium overload, inflammation, and mitochondrial dysfunction, which collectively exacerbate myocardial injury. While several pharmacological and non-pharmacological interventions have shown promise in preclinical models, clinical translation has been limited due to the complexity of human disease and methodological challenges. Current research emphasizes the urgent need for multi-faceted and personalized therapeutic approaches that integrate advanced technologies such as gene therapy, stem cell-based interventions, and nanomedicine. By bridging the gap between bench and bedside, future strategies have the potential to significantly improve myocardial salvage, reduce infarct size, and enhance long-term cardiac function following reperfusion injury. Ultimately, a deeper understanding of MIRI mechanisms, coupled with innovative therapeutic designs, will be pivotal in advancing cardiovascular medicine and improving patient survival and quality of life.

## Figures and Tables

**Table 1:** Preclinical models and the investigated therapeutic strategies for myocardial infarction reperfusion injury.

Animal Model	Method of Inducing MI/Reperfusion Injury	Mechanisms Studied	Therapeutic Agents/Interventions	Key Findings	References
Rat (Wistar/SD)	LAD ligation (30–45 min ischemia + reperfusion)	Oxidative stress, apoptosis, inflammation	N-acetylcysteine, melatonin, cyclosporine A	Reduced infarct size, improved cardiac function	[53]
Mouse (C57BL/6)	Temporary LAD occlusion (30 min) + reperfusion	Mitochondrial dysfunction, necroptosis	MitoQ, necrostatin-1, ischemic preconditioning	Decreased ROS, inhibited necroptosis, cardioprotection	[54]
Rabbit	Coronary artery occlusion (30 min) + reperfusion	Endothelial dysfunction, calcium overload	Nitric oxide donors, verapamil	Improved endothelial function, reduced calcium overload	[55]
Pig	LAD occlusion (60 min) + reperfusion	Microvascular obstruction, no-reflow phenomenon	Adenosine, therapeutic hypothermia	Reduced no-reflow area, improved myocardial perfusion	[56]
Dog	Coronary occlusion + reperfusion (variable duration)	Complement activation, inflammation	Pexelizumab (anti-C5 antibody)	Decreased complement-mediated injury	[57]
Guinea pig	LAD ligation + reperfusion	Arrhythmias, calcium handling	Beta-blockers, calcium channel blockers	Reduced arrhythmias, stabilized calcium homeostasis	[58]

**Table 3:** Key MIRI-associated genes ranked by score.

Rank	Name	Score
1	*TIMP1*	10
2	*SERPINE1*	8
3	*APOB*	7
4	IL1R2	5
4	IL10RA	5
6	CTLA4	4
6	FGG	4
6	FAM20C	4
6	PCSK9	4
6	ANXA2	4

**Table 4:** Significantly enriched pathways associated with the genes identified in MIRI.

Enrichment FDR	No. of Genes	Pathway Genes	Fold Enrichment	Pathways
5.7 × 10^−8^	6	50	50.7	Cholesterol metabolism (https://www.kegg.jp/kegg-bin/show_pathway?hsa04979) (accessed on 17 April 2025)
2.0 × 10^−5^	4	41	41.2	Bladder cancer (https://www.kegg.jp/kegg-bin/show_pathway?hsa05219) (accessed on 17 April 2025)
2.3 × 10^−4^	3	31	40.9	Antifolate resistance (https://www.kegg.jp/kegg-bin/show_pathway?hsa01523) (accessed on 17 April 2025)
2.9 × 10^−3^	2	22	38.4	Arginine biosynthesis (https://www.kegg.jp/kegg-bin/show_pathway?hsa00220) (accessed on 17 April 2025)
1.3 × 10^−10^	9	100	38	AGE–RAGE signaling pathway in diabetic complications (https://www.kegg.jp/kegg-bin/show_pathway?hsa04933) (accessed on 17 April 2025)
3.5 × 10^−5^	4	49	34.5	Malaria (https://www.kegg.jp/kegg-bin/show_pathway?hsa05144) (accessed on 17 April 2025)
3.2 × 10^−4^	3	37	34.2	African trypanosomiasis (https://www.kegg.jp/kegg-bin/show_pathway?hsa05143) (accessed on 17 April 2025)
4.0 × 10^−7^	6	76	33.3	Pertussis (https://www.kegg.jp/kegg-bin/show_pathway?hsa05133) (accessed on 17 April 2025)
9.3 × 10^−11^	10	138	30.6	Fluid shear stress and atherosclerosis (https://www.kegg.jp/kegg-bin/show_pathway?hsa05418) (accessed on 17 April 2025)
4.7 × 10^−3^	2	29	29.1	Linoleic acid metabolism (https://www.kegg.jp/kegg-bin/show_pathway?hsa00591) (accessed on 17 April 2025)

## Data Availability

No new data were created or analyzed in this study. Data sharing is not applicable to this article.
